# Research on health education and health promotion during the process of schistosomiasis elimination III new approaches for student health education

**DOI:** 10.1371/journal.pntd.0013388

**Published:** 2025-08-06

**Authors:** Jing Zhang, Dandan Lin, Fei Hu, Dong Li, Junjiang Chen, Hua Xie, Yifeng Li, Sheng Ding

**Affiliations:** Jiangxi Provincial Institute of Parasitic Diseases, Nanchang, Jiangxi, P.R. China; Jiangsu Institute of Parasitic Diseases, CHINA

## Abstract

Schistosomiasis remains a critical public health challenge in endemic regions, particularly among school-aged children. Despite global efforts, conventional health education approaches show limited success in translating knowledge into sustained practices change. This study evaluates the efficacy of two innovative educational approaches—curriculum-integrated infiltration and stepwise progressive approaches—compared to traditional methods in enhancing schistosomiasis-related knowledge, attitudes, and practices (KAP) among students. A school-based intervention was conducted in Duchang County’s Zhouxi township, with is an area afferted by schistosomiasis in China. Sixth-grade students (n ≈ 300) were divided into three groups: a traditional intervention group receiving standard WHO-aligned lectures, an infiltration group with cross-disciplinary curriculum integration, and a stepwise group with modular, tiered content. KAP outcomes were assessed via validated questionnaires at baseline and post-intervention. Both intervention groups demonstrated significant knowledge gains compared to traditional intervention (post-intervention accuracy: infiltration 89.67%, stepwise 91.10%, traditional intervention 86.50%; *P* < 0.001). Practices knowledge showed the most significant improvement (41.47% increase in the infiltration group vs. 22.38% in traditional intervention). The stepwise approaches achieved the highest overall accuracy (91.10%) but showed no statistically significant advantage over the infiltration approach (*P* > 0.05). Attitudinal improvements were consistent across groups, with high baseline rates limiting further gains (post-intervention: 95.60–96.68%). Curriculum-integrated and stepwise approaches effectively address the knowledge-practices gap in schistosomiasis education. The infiltration strategy, requiring minimal resources, is ideal for practices reinforcement in low-resource settings, while the stepwise approach suits rapid knowledge dissemination in well-resourced areas. These findings advocate for context-adaptive, multisectoral frameworks to optimize school-based interventions, aligning with WHO goals for neglected tropical disease elimination.

## Author summary

Schistosomiasis remains a persistent public health challenge, particularly among school-aged children in endemic regions. Conventional health education approaches often fail to translate knowledge into sustained practices change. This study evaluated two innovative educational approaches—curriculum-integrated infiltration and stepwise progressive approaches—against traditional methods in a schistosomiasis-endemic area near Poyang Lake, China. Sixth-grade students were divided into traditional intervention, infiltration, and stepwise intervention groups. Validated questionnaires assessed knowledge, attitudes, and practices (KAP) pre- and post-intervention. Both intervention groups outperformed traditional intervention in knowledge gains, with practices knowledge showing the most significant improvement. Attitudinal improvements were limited due to high baseline rates. The infiltration approaches, requiring minimal resources, proved ideal for practices reinforcement in low-resource settings, while the stepwise approach excelled in rapid knowledge dissemination. Findings advocate for context-adaptive, multisectoral frameworks to bridge the knowledge-practices gap, aligning with WHO goals for neglected tropical disease elimination. This study provides actionable insights for optimizing school-based interventions in endemic regions globally.

## Introduction

Schistosomiasis, caused by trematode parasites of the genus *Schistosoma*, represents a neglected tropical disease with endemic persistence in tropical and subtropical regions worldwide. This parasitic infection is predominantly transmitted through dermal or mucosal contact with freshwater contaminated by cercariae, the free-swimming larval stage of the parasite. The disease imposes substantial public health burdens, particularly among school-aged children in endemic areas where frequent water contact occurs during daily activities and recreational exposure [1].

Despite sustained global control initiatives spanning decades, including mass drug administration and ecological modifications, residual transmission hotspots persist, underscoring the imperative for innovative practices interventions to complement existing strategies [2]. Within integrated control frameworks, health education has emerged as a critical component for bridging the implementation gap between biomedical approaches and community participation through knowledge enhancement, attitude modification, and preventive practices promotion [3].

China’s national schistosomiasis control program has incorporated health education as a cornerstone intervention since the 1990s, demonstrating particular efficacy in aquatic ecosystems such as the Poyang Lake region characterized by interconnected lakes, marshes, and waterways [4,5]. However, evaluations of conventional health education approaches have revealed limitations in their reliance on passive information delivery through didactic lectures or printed materials, manifested by suboptimal practices adoption and unsustained practice adherence [6,7]. These persistent challenges highlight the urgent requirement for targeted educational innovations specifically designed for high-risk demographic groups, particularly school-aged children who constitute both a vulnerable population and potential agents of long-term practices change [6,8].

School-based interventions have demonstrated global efficacy in enhancing knowledge, attitudes, and practices (KAP) regarding schistosomiasis prevention. For instance, teacher-led health education initiatives in Brazil significantly improved students’ comprehension of disease transmission mechanisms [9], while Tanzanian programs employing participatory approaches successfully promoted improved hygiene practices among participants [10]. In China, school-based health education has been instrumental in reducing schistosomiasis prevalence, particularly in high-risk regions such as Poyang Lake. Although standardized curricula delivered by healthcare professionals have proven effective in enhancing students’ theoretical understanding of schistosomiasis, epidemiological data reveal limited success in sustaining practices compliance, particularly regarding avoidance of contaminated water exposure [4], highlighting the discrepancy between knowledge and action. This persistent knowledge-action discrepancy persists across endemic regions, as evidenced by African studies documenting continued high-risk practices (e.g., recreational water contact and domestic water collection) among students who demonstrate accurate risk perception [6,11]. These collective findings underscore the critical limitation of conventional health education paradigms: while effectively transmitting biomedical knowledge, they exhibit limited capacity to overcome socio-cultural determinants of risk practices in endemic communities.

Emerging evidence underscores the importance of innovative participatory approaches tailored to local contexts in schistosomiasis prevention. In Brazil, integration of schistosomiasis education into art and science curricula through teacher training programs demonstrated greater efficacy in promoting preventive practices among students compared to traditional lecture-based approaches [9]. The Philippines’ “Magic Glasses” initiative achieved improved knowledge retention through augmented reality (AR) visualization of parasite transmission dynamics [12]. China has implemented multifaceted educational interventions targeting primary and secondary students in endemic regions, including: (i) schistosomiasis prevention courses combined with incentive mechanisms and extracurricular activities, (ii) audiovisual education integrated with skills training and reward systems, (iii) situational education, and (iv) contextual education approaches. These strategies significantly enhanced knowledge acquisition and practices compliance while effectively reducing infection rates [13–16]. These successful cases align with the World Health Organization’s recommendations for adaptive educational frameworks [2,17]. Nevertheless, implementation challenges persist, particularly in resource-limited settings. Inadequate teacher preparation and insufficient access to multimedia technologies frequently constrain the scalability of innovative educational approaches in low-income regions [18].

This study evaluated three health education approaches (traditional, infiltration, and stepwise) implemented among schoolchildren in a schistosomiasis-endemic area of China. Through a comparative analysis of the approaches’ impacts on KAP outcomes, we aimed to identify scalable strategies for optimizing school-based schistosomiasis interventions, providing empirical support for localized health education strategies. The findings provide critical evidence to inform the development of China’s schistosomiasis elimination roadmap [19] and contribute to global efforts in addressing the persistent knowledge-practices gap in schistosomiasis control.

## Methods

### Ethics statement

The study protocol received formal approval from the Medical Ethics Committee of the Jiangxi Provincial Institute of Parasitic Diseases (Approval No.: 2021–001). The research team conducted all procedures in strict compliance with the committee’s ethical guidelines and regulatory requirements.

Written informed consent was obtained from the legal guardians of all participating students prior to enrollment. The content and methods of this study were approved by the school headmaster and participating teachers. During the investigation, students were informed of the study’s purpose, procedures, and their right to withdraw at any time. All data involving minors were anonymized (e.g., names removed, age details obscured) and securely destroyed after study completion in accordance with ethical requirements.

### Participants & intervention approaches

This school-based intervention was conducted in a schistosomiasis-endemic area near Poyang Lake, Jiangxi Province. The school is located in Zhouxi Township, Duchang County, one of the areas in the province with the most severe schistosomiasis. The school provides a service to multiple communities and enrolls school-age children from neighboring villages, all of whom are exposed to contaminated water. Prior to the implementation of this study, schistosomiasis prevention and control in this region mainly relied on population screening and treatment alongside routine health education. We decided to split our Sixth-grade students into three study groups according to their class allocation:

- *Traditional intervention group* (Classes 1 & 5, n≈100): The schistosomiasis control education program was implemented through weekly 40-minute instructional sessions conducted by certified professionals from a schistosomiasis prevention and control center. These standardized sessions utilized officially approved schistosomiasis prevention materials developed by the national disease control authority, following the evidence-based health education protocol established by the World Health Organization. Highlights include: (i) transmission mechanisms; (ii) preventive practices; and (iii) treatment policies.
- *Infiltration intervention group* (Classes 2 & 4, n≈100): Implemented cross-disciplinary prevention education integrated into multiple subjects. We incorporated fundamental schistosomiasis prevention principles into standard curricular subjects including mathematics, language arts, natural sciences, environmental studies, and visual arts (complete program details are available in S1 Text).
- *Stepwise intervention group* (Classes 3 & 6, n≈100): Delivered progressive prevention content through sequenced modules. The core health messages and schistosomiasis prevention strategies were systematically identified through coordinated efforts between school administrators, volunteer educators, and project team members. Through structured group discussions, participants collaboratively developed a six-tiered health education framework. This hierarchical system incorporated critical design elements including: (i) presentation formats for educational materials, (ii) thematic focus areas, (iii) content specifications, and (iv) communication strategies encompassing linguistic choices and visual representations. The iterative development process yielded a graduated health education curriculum structured along a progressive difficulty continuum (complete program details available in S2 Text).

**Fig 1:**
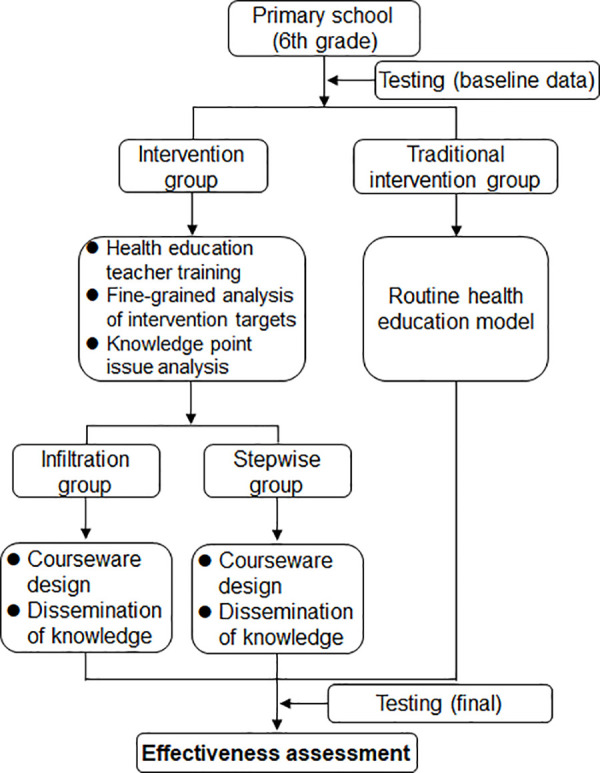
Roadmap for research.

The study took place between October and December 2023. The 11-week intervention protocol (Fig 1) maintained consistent implementation frequency across groups, with weekly sessions matched for duration and scheduling. Classroom teachers received standardized training to ensure protocol adherence, while administrative staff monitored intervention fidelity through routine classroom observations. All participating classes maintained their original student composition throughout the study period. Throughout the study period, the project team members were responsible for teacher training, designing knowledge questionnaires and testing students’ knowledge.

### Schistosomiasis prevention knowledge, beliefs and practices tests

The evaluation framework incorporated a structured questionnaire developed through systematic extraction from the Schistosomiasis Prevention Knowledge Bank. This instrument contained 27 items organized into two conceptual modules: a 20-item knowledge module assessing three competency domains (transmission mechanisms [13 items], protective practices [4 items], and policy awareness [3 items]), accompanied by a 7-item practices module examining perception changes through scenario-based questions. Both modules underwent cognitive pretesting with 15 public health practitioners to ensure content appropriateness. All items employed closed-ended response formats with uniform administration protocols across baseline and post-intervention assessments (complete program details available in S3 Text).

### Statistical analysis

All statistical analyses were performed using SPSS Statistics software (version 27.0; IBM Corp., Armonk, NY, USA). Two-tailed hypothesis tests were implemented with a pre-specified significance level of α = 0.05.

Student performance was evaluated through a standardized assessment, and the average correct rate for both schistosomiasis-related knowledge and attitudes toward disease prevention was calculated for each study group (Equation 1). The normality of data distribution was confirmed using the Shapiro-Wilk test, while homogeneity of variance was verified through Levene’s test. Intergroup comparisons (e.g., traditional intervention group *vs.* infiltration intervention group) and pre-post intervention analyses were conducted using independent samples t-tests. When significant heterogeneity of variance was detected (Levene’s test, *P* < 0.05), Welch’s corrected t-test was employed to ensure robust statistical inference.


$$Average\;correct\;rate\;(\;\%)=\frac{\sum_{n}\left(\frac{Number\;of\;correct\;questions}{Number\;of\;questions\;answered}\times 100\right)}{n}\;(n=\;number\;of\;respondents)$$ (1)

## Results

### Overall correctness

Prior to project implementation, the mean accuracy rates of schistosomiasis prevention knowledge among students in the traditional intervention, infiltration, and stepwise groups were 76.84 ± 11.67%, 75.23 ± 13.04%, and 75.75 ± 11.73%, respectively. Inter-group comparisons revealed no statistically significant differences in baseline knowledge levels (traditional intervention *vs.* infiltration: *t* = 0.886, *P* > 0.05; traditional intervention *vs.* stepwise: *t* = 0.455, *P* > 0.05; infil*t*ration *vs.* stepwise: *t* = 0.094, *P* > 0.05).

Following intervention implementation, significant improvements were observed across all groups, with post-intervention accuracy rates increasing to 86.50 ± 6.57% in the traditional intervention group, 89.67 ± 5.79% in the infiltration group, and 91.10 ± 5.67% in the stepwise group. These post-intervention scores demonstrated statistically significant enhancement compared to baseline measurements in all groups (traditional intervention: *t* = 54.475, *P* < 0.001; infiltration: *t* = 106.524, *P* < 0.001; s*t*epwise: *t* = 139.125, *P* < 0.001).

**Fig 2:**
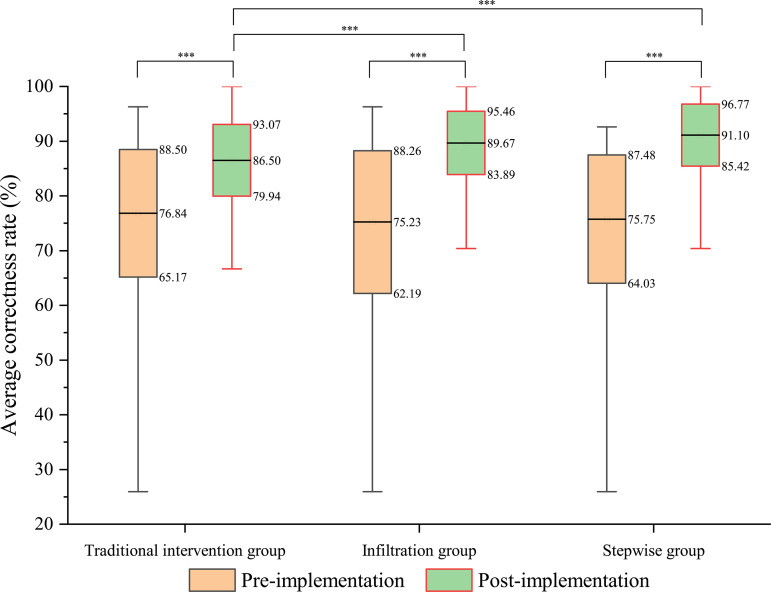
Effect on improving students’ knowledge of schistosomiasis prevention using different health education approaches (*** is *P* < 0.001).

Post-intervention analysis revealed significantly greater knowledge improvement in both intervention groups compared to the traditional intervention group (infiltration *vs.* traditional intervention: *t* = 13.641, *P* < 0.001; stepwise *vs.* traditional intervention: *t* = 28.316, *P* < 0.001). While *t*he stepwise group showed a numerically higher accuracy rate (91.10%) than the infiltration group (89.67%), this difference did not reach statistical significance (*t* = 3.128, *P* > 0.05) (Fig 2).

### Correctness of knowledge points

The baseline mean accuracy rates of schistosomiasis prevention knowledge in the traditional intervention, infiltration, and stepwise intervention groups were 72.08%, 70.63%, and 71.42%, respectively. Following health education implementation, these rates increased to 83.32%, 87.74%, and 89.14%, corresponding to absolute improvements of 15.59%, 24.22%, and 24.81% in the respective groups. Post-intervention comparisons revealed significantly higher knowledge accuracy in both the infiltration group (*t* = 18.424, *P* < 0.001) and stepwise intervention group (*t* = 30.923, *P* < 0.001) compared *t*o the traditional intervention group. However, no statistically significant difference was observed between the infiltration and stepwise intervention groups (*t* = 2.442, *P* > 0.05).

As presented in Table 1, baseline differences were observed in students’ knowledge of schistosomiasis prevention prior to health education implementation. Initial mean correct rates demonstrated distinct variations across knowledge categories: policy knowledge (>85%), basic knowledge (approximately 70%), and practices knowledge (≈60%). Following health education intervention, significant improvements were recorded across all domains, with practices knowledge exhibiting the most pronounced enhancement (22.38% - 41.47% absolute increase). Post-implementation comparisons revealed that the infiltration group achieved significantly higher mean correct rates for both basic knowledge (86.06% *vs.* 81.89%; *t* = 10.553, *P* < 0.01) and prac*t*ices knowledge (84.13% *vs.* 77.64%; *t* = 7.371, *P* < 0.01) compared to *t*he traditional intervention group. While policy knowledge scores in the infiltration group (96.47%) marginally exceeded those of the traditional intervention group (93.59%), this difference did not reach statistical significance (*t* = 2.675, *P* > 0.05). The stepwise interven*t*ion group demonstrated superior performance across all knowledge categories relative to traditional intervention, with mean correct rates of 87.54% for basic knowledge (*t* = 18.932), 85.86% for practices knowledge (*t* = 11.942), and 97.31% for policy knowledge (*t* = 4.607) (*all P* < 0.05). No*t*ably, no sta*t*is*t*ically significant differences emerged between the stepwise and infiltration groups across any knowledge domain (basic: *t* = 1.558; practices: *t* = 0.651; policy: *t* = 0.300; *all P* > 0.05).

**Table 1:** Comparison of the accuracy of three schistosomiasis prevention knowledge points under different health education approaches.

Group	Basic knowledge			Practices knowledge			Policy knowledge		
	Average correctness rate (%)		Change rate (%)	Average correctness rate (%)		Change rate (%)	Average correctness rate (%)		Change rate (%)
	Pre-I	Post-I		Pre-I	Post-I		Pre-I	Post-I	
Traditional intervention group	69.73	81.89	17.44	63.44	77.64	22.38	87.42	93.59	7.06
Infiltration group	68.53	86.06	25.58	59.47	84.13	41.47	87.38	96.47	10.40
Stepwise group	69.18	87.54	26.54	60.85	85.86	41.10	88.37	97.31	10.12

Pre-I: pre-implementation; Post-I: post-implementation.

### Attitudinal correctness of belief practices

**Fig 3:**
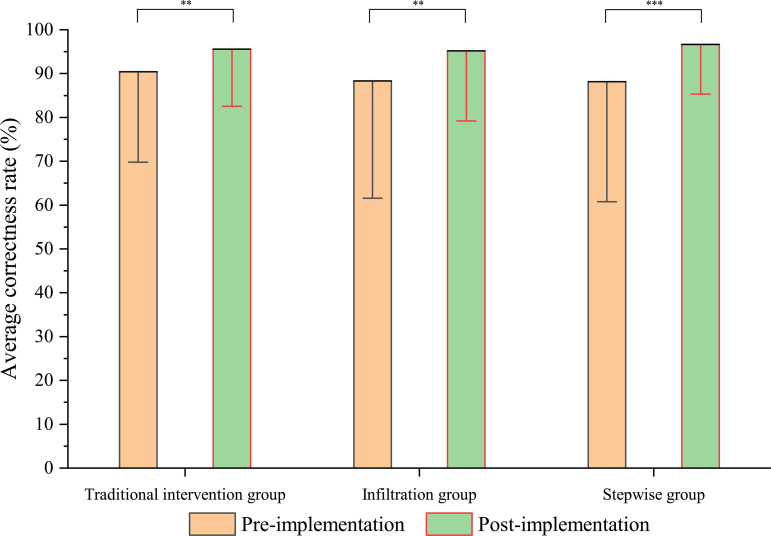
Comparison of different health education approaches to improve the accuracy of beliefs (** is *P* < 0.01; *** is *P* < 0.001).

Prior to the health education interventions, baseline mean accuracy rates for schistosomiasis prevention -related knowledge and belief practices were notably high across all study groups: 90.43% (traditional intervention group), 88.35% (infiltration group), and 88.14% (stepwise group). Initial comparative analysis revealed no statistically significant differences in baseline scores between the intervention modalities (*t* = 0.606, *P* > 0.05). Following the intervention, all three groups demonstrated significant improvements in mean accuracy rates, achieving post-implementation scores of 95.60%, 96.19%, and 96.68% respectively. While intergroup comparisons continued to show no statistically significant differences (*t* = 0.721, *P* > 0.05), wi*t*hin-group analyses revealed substantial increases compared to baseline measurements (traditional intervention: *t* = 10.559; infiltra*t*ion: *t* = 11.233; stepwise: *t* = 18.704; *all P* < 0.05) (Fig 3).

## Discussion

This study demonstrated that both the infiltration and stepwise strategies significantly enhanced students’ schistosomiasis prevention knowledge and promoted more effective preventive practices compared to traditional health education. The progressive knowledge internalization achieved through these tiered approaches likely explains this improvement, contrasting with the passive information delivery characteristic of conventional methods [6,7,20].

Specifically, the infiltration and stepwise approaches proved superior in enhancing practices knowledge. This advantage reflects the greater practical implementation capabilities of interdisciplinary contextualized pedagogy compared to conventional didactics [9]. Our findings corroborate prior research showing traditional school-based health education often improves theoretical knowledge without effectively translating gains into sustained practice changes [21]. This persistent knowledge-practices gap, also observed in settings like Kenya despite extensive education [6], highlights a global public health challenge [6,11]. The effectiveness of the infiltration approach likely stems from its strategic embedding of health messages within students’ daily learning contexts, mirroring successful Brazilian initiatives integrating health education into art and science curricula [9]. The stepwise approach, stratifying health information according to learners’ cognitive capacities, proved particularly effective for diverse student populations. Its systematic segmentation and iterative reinforcement likely contributed to its significant improvement in basic knowledge acquisition (26.54% increase vs. traditional, Table 1), aligning with WHO’s adaptive education framework for NTDs [17]. However, implementing stepwise approaches necessitates robust local partnerships involving educators and community stakeholders [10]. While technology-driven interventions like The Magic Glasses project show promise [12], the stepwise methodology offers advantages in resource-limited settings, though its scalability depends on institutional support and community engagement [10].

A fundamental distinction exists between the two approaches. The stepwise method relies on standardized curriculum development with substantial initial research and development (R&D) investment, making it effective for rapid knowledge dissemination in well-resourced areas. Conversely, the infiltration approach prioritizes teacher autonomy but demands advanced competencies in interdisciplinary integration, proving more conducive to sustaining practice modifications where skilled teaching staff exist.

Resource constraints, including insufficient teacher training and limited access to multimedia technologies, remain systemic barriers globally [18]. While the participatory design of the stepwise approach enhances adaptability, implementation challenges in rural settings (like those observed in Tanzania [10]) underscore the critical need for multisectoral cooperation, exemplified by initiatives like Jiangxi’s Community Resource Integration [22] which leverages cultural practices for health education.

This study also revealed a notable ceiling effect in attitude improvement, likely due to high baseline correctness rates (88.14%-90.43%). This contrasts sharply with settings like rural Côte d’Ivoire where lower pre-intervention awareness allowed dramatic improvements [7]. Such disparities highlight the need for context-specific strategies. Evidence from Hubei Province links negative attitudes to high-risk practices [23], necessitating targeted psychological interventions. Conversely, in regions like Jiangxi where awareness approaches saturation, priorities should shift to enhancing protocol dissemination, optimizing knowledge retention, and systematically reinforcing existing practices through sustained engagement.

Successful implementation of the stepwise approach critically depended on intersectoral collaboration between health and education institutions. Health specialists provided technical expertise for curriculum design while education managed resource allocation, effectively addressing institutional limitations through joint capacity-building (e.g., workshops). This synergy aligns with successful teacher-led interventions internationally, like Brazil’s program sustained through updated materials and expert support [9]. However, challenges persist in rural China, including ambiguous accountability and suboptimal communication. To enhance replicability, we propose institutionalizing collaboration through: (i) Permanent platforms (e.g., Regional Committees) formalizing roles (health: content standardization; education: implementation); (ii) Codified protocols (e.g., Collaboration Guidelines); and (iii) Biannual joint training. Combining medical expertise with pedagogical competence appears fundamental to sustaining quality.

The findings carry significant policy and practical implementation. China’s National Roadmap for Schistosomiasis Elimination [19] could be strengthened by integrating these evidence-based approaches into comprehensive strategies emphasizing teacher empowerment and community engagement. Prioritizing professional development for context-adapted curriculum delivery is crucial. Furthermore, interventions combining targeted health education with psychosocial support are needed for vulnerable populations, such as rural left-behind children facing compounded risks [24].

Future schistosomiasis control programs should adopt a tiered adaptation strategy: deploying the stepwise approach in high-risk areas for rapid knowledge barriers and the infiltration approach in low-risk regions to consolidate practices. A tripartite collaboration framework (health, education, community) is recommended. Strengthening parental engagement (e.g., community lectures) could address school-centric limitations. Additionally, leveraging digital tools (e.g., low-cost AR modules) could overcome resource bottlenecks and standardize content delivery.

## Limitations

While this study provides valuable insights, several limitations should be acknowledged. First, the intervention was implemented exclusively at a school within the Poyang Lake region. This geographic specificity may limit the generalizability of the findings to populations in distinct socio-cultural contexts or ecological settings, such as mountainous regions or urban centers. Second, the three-month evaluation period restricted our ability to assess longitudinal outcomes. Consequently, no definitive conclusions can be drawn regarding the durability of observed practices changes over extended periods. Future investigations incorporating multi-regional sampling and extended follow-up durations would enhance the evidence base for developing context-adaptive schistosomiasis education programs.

## Conclusions

In conclusion, both innovative models significantly outperformed traditional education in improving schistosomiasis knowledge, with the infiltration approach excelling in behavioral reinforcement and stepwise approaches achieving highest overall accuracy. To optimize impact, resource-limited regions should prioritize infiltration integration into core curricula, while endemic hotspots deploy stepwise modules. National elimination programs must institutionalize teacher training and multisectoral collaboration to sustain these gains.

## Supporting information

S1 Text Schedule for infiltration schistosomiasis health education courses. (PDF)

S2 Text Content of stepwise health education interventions. (PDF)

S3 Text Questionnaire on schistosomiasis prevention knowledge. (PDF)

S1 Data List of raw questionnaire data. (XLSX)
