# Supplementary material for: Research on health education and health promotion during the process of schistosomiasis elimination III new approaches for student health education
Source: PLoS Negl Trop Dis. 2025 Aug 6;19(8):e0013388. doi: 10.1371/journal.pntd.0013388 (PMC12338806; doi:10.1371/journal.pntd.0013388)
Supplement: S1 Text — (PDF) [file pntd.0013388.s001.pdf]

Schedule for infiltration schistosomiasis health education courses

Timetable	Teaching content
1st week	**Maths lessons:** Teaching content (the meaning of ratio) - Infiltration of knowledge: recognising the size of a snail and the grassy island around Zhouxi Town.
2nd week	**Language and literature lessons:** Teaching content (grasslands) - Infiltration of knowledge: getting to know nature(getting to know the grassland at Poyang Lake, getting to know the lake and grassland in our hometown of Zhouxi Town in Duchang County, getting to know the nail snails in the grassland and schistosomes in the lake water).
3rd week	**Maths lessons:** Teaching content (mixed operations with fractions) - Infiltration of knowledge: incorporate calculating the area of grassy continents, the area of snail eradication, and the amount of medication used for treatment into application questions.
4th week	**Maths lessons:** Teaching content (percentages) - Infiltration of knowledge: environmental snail eradication, pasture closure, cattle and sheep confinement.
5th week	**Maths lessons:** Teaching content (fan chart) - Infiltration of knowledge: management of the snail area and control of schistosomiasis transmission.
6th week	**Science lessons:** Teaching content (big search for organisms, distribution maps of organisms, etc.) - Infiltration of knowledge: awareness of organisms around the town of Zhouxi: grassy continents, distribution of snails, cows, sheep and pigs.
7th week	**Science lessons:** Teaching content (plants and animals and interrelationships) - Infiltration of knowledge: animals (cows, sheep, pigs + grassy continents + snails, transmission of schistosomiasis).
8th week	**Art lesson:** Teaching content (drawing of objects) - Infiltration of knowledge: drawings of snails and *schistosomes*, and drawings of the life history of *schistosomes* and warning signs to prevent *schistosome* infection.
9th week	**Science lessons:** Teaching content (observing the world of life around us through a microscope) - Infiltration of knowledge: observing the morphology of miracidia, cercariae and adult schistosomes using a microscope.
10th week	**Language and literature lessons:** Teaching content (growing up in summer) - Infiltration of knowledge: the metamorphosis and growth of *schistosoma* over 30 days in summer.

Key messages for control：

I. Basic knowledge of schistosomiasis control: 1) That there is an endemic disease (schistosomiasis) in our hometown and which places are danger zones; 2) Knowing the dangers that schistosomiasis can cause to the body; 3) Knowing how schistosomiasis is transmitted (human and animal faecal transmission) and which months of the year are the high risk seasons; 4) Knowing that the 'nail snail' is an intermediate host of *schistosoma*.

II. Healthy behaviours and lifestyles: No contact with infected water, no faecal matter in water, keeping cattle in captivity (using machines instead of cattle), cooperating with doctors for schistosomiasis screening and treatment, using safe water and sanitary toilets.

III. Health skills for schistosomiasis control: 1) Protect yourself or your family members if you have to come into contact with infected water (rubber shoes, rubber clothes, rubber gloves, etc.); 2) Learn to differentiate between the symptoms of acute schistosomiasis and the symptoms of common fever and diarrhoea; 3) Learn to read the schistosomiasis advertisements and warning signs.
