# Supplementary material for: Research on health education and health promotion during the process of schistosomiasis elimination III new approaches for student health education
Source: PLoS Negl Trop Dis. 2025 Aug 6;19(8):e0013388. doi: 10.1371/journal.pntd.0013388 (PMC12338806; doi:10.1371/journal.pntd.0013388)
Supplement: S2 Text — (PDF) [file pntd.0013388.s002.pdf]

Content of stepwise health education interventions

Stage	Timetable	Topic	Health education content	Key messages for control
1	1st week	● Getting to know the body<br>● Develop healthy and hygienic habits	1. Don't drink raw water, drink clean water.<br>2. Don't wash hands with dirty water, wash hands with tap water.<br>3. Don't urinate or defecate outdoors, use toilets at home (school).	Daily behavior habits; do not play with water near the lake.
2	3rd week	● Getting to know doctors<br>● Health and Body	1. What is a doctor? Why do people get sick?<br>2. Some diseases are caused by eating unclean things, some by contact with unclean water, and some by parasites in the soil that spread disease.<br>3. What is a person's normal body temperature? When should a person go to the doctor? (Tell your parents if you have a headache, fever, diarrhea, or if you feel unwell that you should go to the doctor, and cooperate with the doctor's examination).	Reinforce the daily hygiene and health habits that have been developed; reinforce not going to the lake to play in the water.
3	5th week	Environment and health (understanding schistosomiasis)	1. know the village they live in and its surroundings.<br>2. to know the risk factors and disease causing risk factors in the environment where they live.<br>3. learn to recognise the danger signs for schistosomiasis control and understand the content of the slogans and posters for schistosomiasis control.	Get to know Poyang Lake, Grassy Island, cattle, sheep, snails, schistosomiasis and do not play in the water where schistosomiasis occurs.
4	7th week	Life (production) and health	1. How is schistosomiasis transmitted?<br>2.Health risks, precautionary awareness and behaviours when living at Lake Poyang.<br>3.Occupational health risks for parents (fishing boat people).	Sources of schistosomiasis infection, routes of transmission; production risks for people living on fishing boats.
5	9th week	Disease and health (control and treatment)	1. Healthy lifestyles and modes of production.<br>2. How to prevent and control schistosomiasis?<br>3. Symptoms of schistosomiasis?<br>4. How to treat schistosomiasis?	Manure management;<br>Safe water;<br>Protective measures in production and living.
6	11th week	Community and health	1. Self-healthy choices: how to protect yourself and maintain a healthy body.<br>2. Teaching their parents and relatives about health knowledge, skills and attitudes for schistosomiasis control.	Formation of a self-concept of health; transfer of health knowledge and concepts of schistosomiasis control to the family.
