# Supplementary material for: Research on health education and health promotion during the process of schistosomiasis elimination III new approaches for student health education
Source: PLoS Negl Trop Dis. 2025 Aug 6;19(8):e0013388. doi: 10.1371/journal.pntd.0013388 (PMC12338806; doi:10.1371/journal.pntd.0013388)
Supplement: S3 Text — (PDF) [file pntd.0013388.s003.pdf]

# Questionnaire on schistosomiasis prevention knowledge for primary and secondary school students

Name: ______ Genders: ______ Age: ______

**1. Knowledge of schistosomiasis prevention (single choice)**

1.1 Did you know about schistosomiasis?

A. Yes; B. No

1.2 Which of the following animals that live near the water can transmit schistosomiasis?

A. Frogs; B. Oncomelania hupehensis; C. Winkle mollusk

1.3 Can schistosomes be present in dew on grass where nail snails are found?

A. Yes; B. No

1.4 How is schistosomiasis contracted?

A. Breathing in stale air; B. Exposure to water contaminated by Schistosoma parasites; C. Eating food that has been crawled on by flies

1.5 How do schistosomes enter the human body?

A. Inhaled through the nostrils; B. Ingested orally; C. Penetrating through the skin

1.6 What are the symptoms of schistosomiasis?

A. Increased heart rate; B. Fever, abdominal pain, diarrhea; C. Cough

1.7 What are the consequences of a severe case of schistosomiasis?

A. Loss of ability to work; B. Inability of women to conceive; C. Both of the above

1.8 What is the purpose of the government's promotion of closed continents for grazing and the confinement of cattle and sheep in schistosomiasis-endemic areas?

A. Active development of animal husbandry; B. Control of schistosomiasis transmission

1.9 What will happen to anyone who damages or removes a schistosomiasis control warning sign, such as one indicating a snail-infested area, without authorization?

A. Economic penalties; B. No penalties will be imposed

1.10 Does the area where the Schistosomiasis Control Prohibition sign is located have the highest risk of schistosome infection?

A. Yes; B. No

1.11 Which of the following behaviors do you think could lead to you contracting schistosome?

A. Exposure to water contaminated by the parasite Schistosoma haematobium; B. Contact with patients with schistosomiasis or diseased cattle

1.12 What is the easiest and most effective way to prevent getting schistosomiasis?

A. No contact with patients; B. No contact with faeces C. No contact with water contaminated with Schistosoma haematobium

1.13 If a cow or sheep contracts schistosomiasis, can the disease be transmitted through its faeces?

A. Yes; B. No

**2. Schistosomiasis prevention attitudes beliefs behaviors (single choice)**

2.1 Do you swim and play in the river in the summer?

A. Yes; B. No

2.2 Do you go to the schistosomiasis control classes at school?

A. Yes; B. No

2.3 Do you discourage other students when they go to play in the water?

A. Yes; B. No

2.4 Would you advise your family members to take precautions when doing water-based activities such as fishing?

A. Yes; B. No

2.5 What do you do when you are examined and treated for schistosomiasis?

A. Proactive; B. Passive; C. Refuse

2.6 Would you be willing to take the medication as required by your doctor if you were diagnosed with schistosomiasis?

A. Yes; B. No

2.7 In your opinion, is schistosomiasis dangerous to the body?

A. No harm; B. Some harm; C. A lot of harm

**3. Judgement questions (√ if correct, × if incorrect)**

3.1 You cannot catch schistosomiasis by shaking hands with an infected person. (√)

3.2 Anyone can be infected with schistosomes. (√)

3.3 You cannot contract schistosomiasis by simply swimming in water containing snails (epidemic water). (×)

3.4 Schistosome infections are most prevalent from April to October. (√)

3.5 Once you have been cured of schistosomiasis, you will not contract the disease again if you come into contact with infected water. (×)

3.6 It is safe for cattle to graze on grassy continents where snails breed, as this does not transmit schistosomiasis. (×)

3.7 You can’t get schistosomiasis from playing on the grassy island outside the embankment in the summer, as long as you don’t come into contact with infected water. (×)
